# DNA Methylation Dynamics in Response to Drought Stress in Crops

**DOI:** 10.3390/plants13141977

**Published:** 2024-07-19

**Authors:** Xiaolan Rao, Shengli Yang, Shiyou Lü, Pingfang Yang

**Affiliations:** State Key Laboratory of Biocatalysis and Enzyme Engineering, School of Life Sciences, Hubei University, Wuhan 430062, China; xiaolan.rao@hubu.edu.cn (X.R.); shengliyang1998@outlook.com (S.Y.); shiyoulu@hubu.edu.cn (S.L.)

**Keywords:** epigenetics, DNA methylation, drought stress response

## Abstract

Drought is one of the most hazardous environmental factors due to its severe damage on plant growth, development and productivity. Plants have evolved complex regulatory networks and resistance strategies for adaptation to drought stress. As a conserved epigenetic regulation, DNA methylation dynamically alters gene expression and chromosome interactions in plants’ response to abiotic stresses. The development of omics technologies on genomics, epigenomics and transcriptomics has led to a rapid increase in research on epigenetic variation in non-model crop species. In this review, we summarize the most recent findings on the roles of DNA methylation under drought stress in crops, including methylating and demethylating enzymes, the global methylation dynamics, the dual regulation of DNA methylation on gene expression, the RNA-dependent DNA methylation (RdDM) pathway, alternative splicing (AS) events and long non-coding RNAs (lnc RNAs). We also discuss drought-induced stress memory. These epigenomic findings provide valuable potential for developing strategies to improve crop drought tolerance.

## 1. Introduction

Drought poses a significant threat to global agricultural productivity and crop quality [1]. Climate change has exacerbated the scale, frequency, and intensity of drought [2]. Based on reported extreme events from 1964 to 2007, a single drought led to an average loss of 10% in cereal production [3]. In China, climate-related disasters such as droughts and floods have caused damage to rice, maize, soybean and wheat yields ranging from 4.5% to 11.6% during the period of 1982–2012 [4]. However, plants are sessile and unable to escape from adverse circumstances, which may persist throughout the plant’s lifecycle and that of its offspring. To survive current environmental stresses and potential future assaults, plants have evolved a variety of genetic and epigenetic regulatory mechanisms to enhance resistance and memorize the experience.

**Figure 1:**
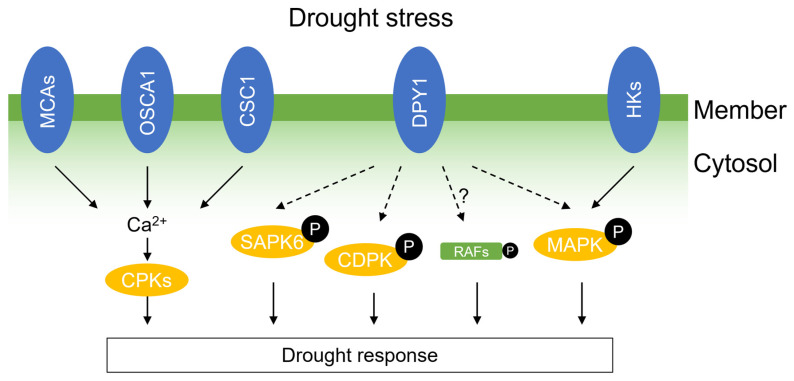
Osmosensors mediate drought stress signaling. MID1-COMPLEMENTING ACTIVITY (MCAs), REDUCED HYPEROSMOLALITY-INDUCED CA2+ INCREASE1 (OSCA1), and CALCIUM PERMEABLE STRESS-GATED CATION CHANNEL 1 (CSC1) are Ca2+ channels that become activated in response to drought stress, leading to an increase in cytosolic calcium ions (Ca2+). Calcium-dependent protein kinases (CPKs) sense the elevated Ca2+ levels in the cytosol and initiate downstream signaling cascades. Under drought stress, DROOPY LEAF1 (DPY1) is phosphorylated, resulting in the global phosphorylation of various proteins, including STRESS ACTIVATED PROTEIN KINASE6 (SAPK6), mitogen-activated protein kinases (MAPKs), calcium-dependent protein kinases (CDPKs), and Raf-like kinases (RAFs). Histidine kinases (HKs) located on the plasma membrane also participate in the MAPK signaling pathway.

Drought stress leads to a decrease in cell turgor, resulting in changes in cell wall composition and structure, as well as damage to the plasma membrane and cell wall [5,6]. Osmosensors, such as Histidine kinases (HKs), DROOPY LEAF1 (DPY1), the osmosensitive Ca^2+^ channels REDUCED HYPEROSMOLALITY-INDUCED CA^2+^ INCREASE1 (OSCA1), CALCIUM PERMEABLE STRESS-GATED CATION CHANNEL 1 (CSC1) and MID1-COMPLEMENTING ACTIVITYs (MCAs), perceive the effects of drought stress on plant cells [5,6,7,8] (Figure 1). Subsequently, various signaling pathways, including calcium signaling, ABA-dependent and ABA-independent signaling, and reactive oxygen species (ROS) are activated [6,9,10]. Multiple regulatory processes at the transcriptional, post-transcriptional, post-translational, and epigenetic levels are integrated in the plant’s response to drought. The molecular mechanisms of drought stress perception and signaling pathways have been comprehensively reviewed in recent publications [6,9,11,12,13].

Along with the significant advancements in understanding the genetic basis of abiotic stress signaling and responses in plants, there has been a growing focus on the intricate epigenetic regulatory mechanisms triggered by abiotic stresses. Epigenetic regulation involves three main events: DNA methylation, histone post-translational modifications and RNA-mediated gene silencing [14]. Methylation on the fifth position of the pyrimidine ring of cytosine (5-methylcytosine, 5 mC) or the sixth position of the purine ring of adenine (N^6^-methyladenosine, 6 mA) have been frequently detected as epigenetic markers in eukaryotes [15,16,17]. In plants, methylation of cytosine bases predominantly occurs in all three sequence contexts, CG, CHG and CHH (where H refers to A, T or C) [16,18]. DNA methylation primarily maintains genome stability and plays a crucial role in gene regulation in response to environmental factors [19,20,21].

The available methods of DNA methylation analysis, such as Whole-genome Bisulfite Sequencing (WGBS), Methylation-Sensitive Amplification Polymorphism Sequencing (MSAP-Seq) and MethylRAD, have been described in previous reports [22,23]. Among them, WGBS is the gold standard for analyzing dynamic DNA methylomes at single-base resolution. The model plant *Arabidopsis* was the first plant to have its genome sequenced and its whole-genome methylome mapped [24,25,26]. In *Arabidopsis*, the involvement of DNA methylation in response to biotic and abiotic stress has been extensively studied [18,27,28].

Recently, whole-genome methylome analyses have been applied in non-model crops with the availability of high-quality reference genomes. The combination of WGBS and RNA sequencing is an efficient strategy for a comprehensive evaluation of the relationship between methylome and gene expression. In this review, we highlight the current achievements in DNA methylation dynamics in response to drought stress. These recent reports indicate the conservation and divergence of epigenetic strategies in crops exposed to abiotic stress and provide a foundation for genetic manipulation to improve stress resistance in crops.

## 2. DNA Methyltransferases and Demethylases in Drought Tolerance

A dynamic balance between DNA methylation and demethylation through precise regulation is essential for various cellular biological processes. Different enzymes have been characterized to activate or deactivate specific DNA methylation states [28]. In *Arabidopsis*, CG, CHG and CHH cytosine methylation are preferentially catalyzed by METHYLTRANSFERASE 1 (MET1), CHROMOMETHYLASE 3 (CMT3) and DOMAINS REARRANGED METHYLASE 2 (DRM2), respectively [29,30]. DNA demethylation is performed by a family of four DNA glycosylases, including REPRESSOR OF SILENCING 1 (ROS1), TRANSCRIPTIONAL ACTIVATOR DEMETER (DME), DEMETER-LIKE PROTEIN 2 (DML2) and DML3 [31,32]. The nucleosome remodeler DECREASED IN DNA METHYLATION 1 (DDM1) plays a critical role in maintaining DNA methylation of transposable elements [33].

Groups of DNA methyltransferases and demethylases have been identified and functionally characterized in crops [34,35,36,37,38]. In rice, a total of nine DNA methyltransferases (two MET, three CMT, and four DRM) have been identified [34]. The gene numbers in MET and DRM family are duplicated compared with that in *Arabidopsis*. Similarly to their *Arabidopsis* homologues, OsMET1b, OsCMT3a and OsDRM2 are the main methylases for CG, CHG and CHH methylation, respectively [39]. A slight difference was observed in CMT2, which does not affect CHG methylation in rice but maintains both CHH and CHG methylation in *Arabidopsis* [39]. This indicates a conserved mechanism in DNA methylation in *Arabidopsis* and crops.

Gene expression profiling has shown that drought stress alters the transcriptional levels of genes encoding DNA methyltransferases and demethylases [34,35,36,37,38]. A rhythmic expression between DNA methyltransferase and demethylase genes was observed in apple [40] and mulberry [41] under drought stress at different time points, suggesting a coordinated response between DNA methylation and demethylation.

Suppression of DNA methyltransferases may activate the expression of stress response genes [42]. Mutants of *cmt2* and *drm2* in *Arabidopsis* showed increased tolerance to heat stress and cold stress, respectively [43,44]. The application of 5-azacytidine, an inhibitor of DNA methyltransferase, enhanced freezing tolerance in *Arabidopsis* [44]. Similarly, downregulation of the nucleosome remodeler *DDM1* in poplar RNAi lines showed more tolerance to drought-induced cavitation by regulating genes involved in hormone-related stress responses [45]. Cotton plants injected with the DNA methylation inhibitory reagent 5-azacytidine also showed improved drought resistance [46].

## 3. Methylation Landscapes under Drought Stress

The level of DNA methylation varies across plant kingdom [47]. A positive correlation was found between the DNA methylation level (CG and CHG context) and genome size [47,48]. Due to the variation in both the size and level of methylated regions between species, the effect of drought stress on DNA methylation patterns is species-specific. When comparing methylomes under drought stress, the overall methylation level has remained unchanged in *Arabidopsis* [49]; increased in mulberry [41], linseed [50] and *P. trichocarpa* [51]; and significantly decreased in faba bean [52], perennial ryegrass [53] and *P. tomentosa* [54]. Additionally, a higher methylation level induced by drought stress was observed in the drought-sensitive line than that in the drought-tolerant line of rice [55], maize [56], mungbean [57], wheat [58] and *P. tomentosa* [54], suggesting a negative correlation between DNA methylation and drought resistance.

Among the three DNA methylation contexts, the CHH methylation level shows the highest correlation with drought stress in most crops [46,55,59]. However, in mulberry, the CG methylation level shows a higher correlation with drought stress [41]. Furthermore, drought-induced changes in CHH methylation predominantly occurred at TE sequences and/or gene promoter regions [40,49,55,59].

## 4. Correlation between DNA Methylation and Gene Expression

The role of DNA methylation varies in different genomic regions. Methylation near gene promoters typically suppresses gene expression by preventing the binding of transcription factors [23,60]. Nevertheless, there are numerous exceptions that suggest a complex relationship between promoter methylation and gene expression [61,62]. DNA methylation in gene bodies is likely linked to transcript elongation, alternative splicing (see Section 6) and the suppression of repetitive DNA elements [60,63].

Through an integrated analysis of transcriptome and methylome data, a relatively conserved relationship between DNA methylation density and the gene expression level has been observed in *Arabidopsis* and other crops in response to drought stress [27,51,55]. Generally, the gene expression level is positively correlated with the methylation levels in gene bodies (especially in the CG context) and/or distal promoter regions (especially in the CHH context) but negatively correlated with methylation density at the transcription start site (TSS), transcription termination site (TTS) and downstream regions. Hypermethylation of TE regions may contribute to transposon suppression, and the expression level of genes can be affected by nearby TEs [27,51,55].

Differentially methylated regions induced by drought stress play an important role in determining expression levels of stress-responsive genes. Groups of candidate genes, whose expression levels were correlated to DNA methylation, were discovered using both WGBS and RNA-seq. Many of these genes were enriched in stress response pathways such as hormone signaling pathways, transcription factors, programmed cell death, and reactive oxygen species (ROS) synthesis. Specifically, changes in the CHH context under drought stress contributed to higher expression of drought-related transcription factors, including members of *AP2*, *WRKY*, *MYB*, *NAC,* and *bHLH* transcription factor families [40,41,51,55,59,64,65].

Several key genes have been characterized to show an association with DNA methylation, transcriptional expression, and drought tolerance. For example, in maize, an increased level of DNA methylation at the *ZmNAC111* promoter leads to a reduction in its expression and increased drought sensitivity [66]. Methylation at the *HvCKX2.1* promoter is induced by drought stress and accelerates shoot emergence in barley [67]. Additionally, the expression of *MdRFNR1*, a positive regulator of drought tolerance in apple, is induced by methylation on a MITE insertion in its promoter [68].

## 5. The RNA-Dependent DNA Methylation Pathway

**Figure 2:**
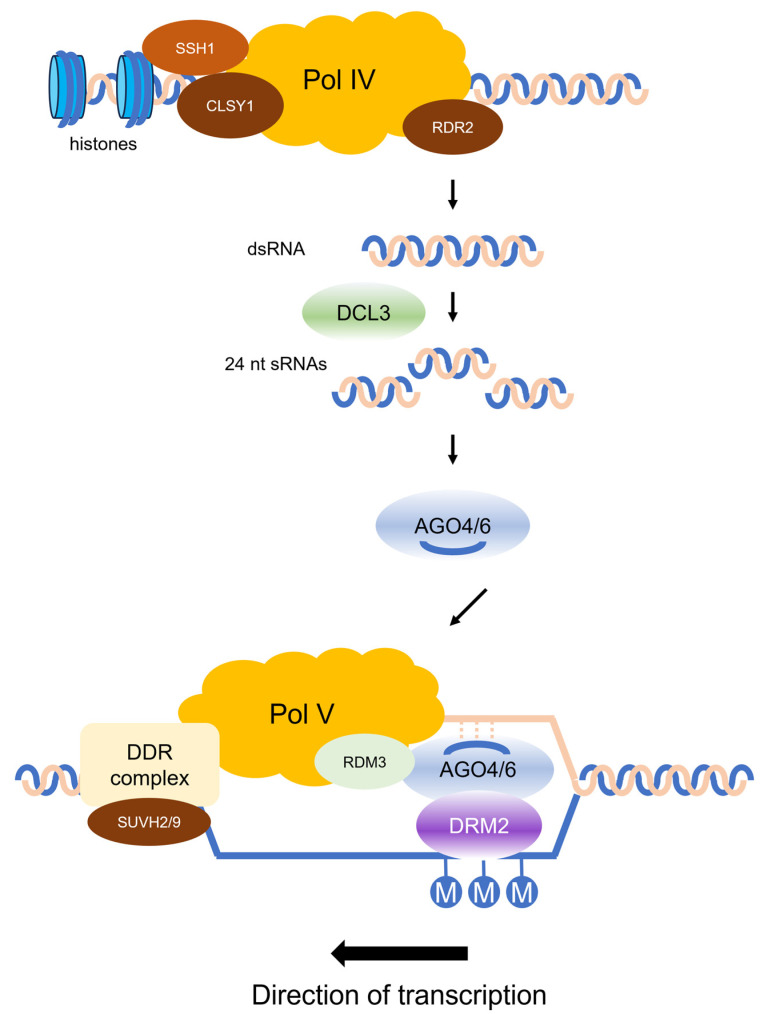
The canonical RNA-directed DNA methylation pathway in Arabidopsis. RNA Polymerase IV (POL IV) interacts with the CLASSY protein (CLSY1) and SAWADEE homeodomain homolog 1 (SHH1), which is bound to dimethylated histone H3 lysine 9 (H3K9me2). POL IV generates single-stranded RNA precursors, and RNA-DEPENDENT RNA POLYMERASE 2 (RDR2) produces double-stranded RNA (dsRNA). DICER-LIKE PROTEIN 3 (DCL3) cleaves the dsRNA into 24-nucleotide small interfering RNAs (siRNAs). The siRNAs bind to ARGONAUTE proteins (AGO4 and AGO6). The AGO-sRNA duplex binds to complementary sequences along an RNA scaffold produced by RNA Polymerase V (Pol V). This reaction is assisted by RDM3, the DDR complex, and SUVH2/9. RDM3 is a putative transcription elongation factor of Pol V. The DDR complex consists of DRD1, DMS3, and RDM1. SUVH2/9 interacts with pre-existing methylated DNA. Domains Rearranged Methyltransferase 2 (DRM2), recruited by AGO-sRNA duplex, catalyzes de novo methylation in nearby DNA.

The RNA-directed DNA methylation (RdDM) pathway is a unique process in plants that non-coding RNA initiates de novo DNA methylation in a sequence-specific manner [28,69]. The canonical RdDM pathway identified in *Arabidopsis* generates 24 small interfering RNAs (siRNAs) that interact with DNA methyltransferases to establish de novo DNA methylation (Figure 2) [28,69]. The siRNA precursors are initially produced by RNA POLYMERASE IV (POL IV) and converted into double-stranded RNA (dsRNA) by RNA-DEPENDENT RNA POLYMERASE 2 (RDR2). The dsRNAs are then split into 24-nt siRNAs by DICER-LIKE PROTEIN 3 (DCL3) and loaded onto ARGONAUTE 4 (AGO4) and AGO6 proteins. Scaffold RNAs, which are transcribed by POL V, are complementary with 24-nt siRNAs and de novo methylated by DRM2 at the target locus. Many accessory proteins are required in the process. POL IV, SAWADEE HOMEODOMAIN HOMOLOGUE 1 (SHH1) and SNF2 DOMAIN-CONTAINING PROTEIN CLASSY 1 (CLSY1) interact with each other. SHH1 binds to dimethylated histone H3 lysine 9 (H3K9me2), and CLSY1 is a chromatin-remodeling protein. RNA-DIRECTED DNA METHYLATION 3 (RDM3) is a transcription elongation factor of POL V and enhances the interaction of AGO4/6 and POL V. The DDR complex, which is assembled by DEFECTIVE IN RNADIRECTED DNA METHYLATION 1 (DRD1), DEFECTIVE IN MERISTEM SILENCING 3 (DMS3) and RDM1, is required for chromatin remodeling to facilitate of POL V. RDM1 also plays a key role in the reaction of de novo DNA methylation through interaction with both AGO4 and DRM2. VARIEGATION 3–9 HOMOLOG PROTEIN 2 (SUVH2) and SUVH9 aid in the recruitment of POL V at the proper location through the recognition of methylated cytosine and interaction with the DDR complex.

RdDM pathway primarily suppresses TE expression through de novo DNA methylation on new TE insertions and maintenance of stable methylation over existing TEs [69,70]. The activity of TEs triggered under abiotic stress is reinforced by the RdDM pathway. For example, one TE family *Rider* retrotransposon in tomato was activated by drought stress and controlled by the RdDM pathway [71]. The RdDM pathway is also associated with methylation over TE elements at the promoter region of *ZmNAC111* in maize [66], *HvCKX2.1* in barley [67], and *MdRFNR1* in apple [68] under drought stress. In rice and maize, changes in siRNA abundance and DNA methylation induced by water deficit showed a positive correlation at different genetic regions [56,72]. These results suggest the involvement of the RdDM pathway in response to drought stress in crops.

## 6. Alternative Splicing Regulated by DNA Methylation under Drought Stress

Recent evidence has demonstrated the role of DNA methylation in the regulation of alternative splicing (AS), partly through changes in methylation status on alternatively spliced exons [73,74]. AS is an important mechanism that enables the generation of multiple transcript isoforms from a single gene [75]. It serves as a fundamental post-transcriptional regulatory process that influences the stability, expression, or subcellular localization of specific isoforms [73]. Approximately 42% to 61% of genes in plants undergo alternative splicing [76]. AS events contribute to the reprogramming of the transcriptome in various aspects of plant development, including seed germination, flowering, and the circadian clock [77,78,79]. In plants, AS also plays a crucial role in acquiring abiotic stress tolerance, particularly in ABA signaling-mediated drought resistance [80].

Genes can undergo two types of AS, classical and alternative *cis*-splicing (termed *cis*-splicing), and genetic and self-*trans*-splicing (termed *trans*-splicing) [81]. AS events are performed by the spliceosomal complex in the nucleus, which is facilitated by several splicing factors such as members of the SERINE/ARGININE (SR) proteins [82]. Three kinases, namely the serine/arginine protein kinases (SRPKs), pre-mRNA processing factor 4 (PRP4Ks) protein kinases, and Arabidopsis FUS3 complement (AFC), have been identified as essential for spliceosome formation and splice site recognition [82,83,84]. The differential expression of these splicing-related kinases in *Arabidopsis*, rice, and wheat under abiotic stress suggests their involvement in stress response [82,83,84].

A higher level of DNA methylation was observed in exon compared to flanking introns [73]. Therefore, the differential distribution of DNA methylation levels in the gene body is considered as a marker for distinguishing exons from introns [73]. DNA methylation can influence both exon selection and alternative splicing of mRNA precursors [73,75]. However, the relationship between DNA methylation and AS events varies among crops under drought stress. In maize, the DNA methylation level at the gene body was positively correlated with AS events but negatively correlated with gene expression, indicating the dual regulation of DNA methylation on gene expression and AS in response to drought stress [56]. In linseed varieties, AS events rapidly accumulated under drought stress but were not associated with gene body methylation dynamics [50]. In *P. trichocarpa*, the relationship between methylation and *cis*-splicing events was not found because methylated sites were absent in the majority of *cis*-splicing genes [51]. This suggests different mechanisms in the regulation of alternative splicing in crops.

However, the current understanding of the association between AS and DNA methylation is limited to second-generation sequencing technologies, which produce short reads and may not accurately predict AS. Recent advancements in third-generation sequencing technology, providing longer reads to directly detect epigenetic modifications of transcriptional isoforms [85,86], will offer valuable insights into the epigenetic regulation of AS under abiotic stresses in crops.

## 7. Long Noncoding RNAs and DNA Methylation under Drought Stress

Long noncoding RNAs (lncRNAs) are a class of RNA transcripts longer than 200 nucleotides that are not translated into functional proteins [87,88]. They can affect the stability of cytoplasmic mRNAs and control different stages of mRNA processing such as splicing, turnover and translation [87,89]. Plant lncRNAs are involved in essential biological processes, including vernalization, fertility, photosynthesis pathways, and responses to biotic/abiotic stresses, based on their tissue- and condition-specific expression patterns [90,91]. Studies have shown that the expression of many lncRNAs is responsive to drought stress in *Arabidopsis* [92], maize [93] and *Morus alba* [94].

Recent research has revealed the interplay between DNA methylation and lncRNAs. The expression levels of lncRNAs are negatively regulated by DNA methylation in all three sequence contexts, both in their flanking and body regions [95]. LncRNAs also play a role in guiding DNA methylation in gene promoters by interacting with DNA methyltransferases, thereby influencing gene expression at the epigenetic level [96].

The involvement of lncRNAs and DNA methylation under drought conditions has been observed in crops. In cotton, lncRNAs may undergo splicing into microRNAs, which regulate methylation in response to drought stress [46]. In rice, lncRNAs and DNA methylation may serve as memory factors in establishing short-term drought memory by activating genes related to drought resilience [97]. However, further research is needed to uncover the detailed mechanism underlying the regulation of epigenetics and lncRNAs.

## 8. Drought Stress Memory

Plants have evolved a mechanism to acquire memorization of environmental experiences, aiding in their adaptation to recurring stress. Epigenetic stress memory is defined as chromatin marks at certain loci that are induced by environmental inputs and transmittable through cell divisions for the same stress in the future [98]. These environment-triggered epigenetic marks include posttranslational modifications of nucleosomal histones (e.g., acetylation, methylation, phosphorylation, and ubiquitination) and DNA methylation or demethylation at the stress-responsive loci [98]. For example, increased histone H3 lysine-4 trimethylation (H3K4me3) and histone H3 lysine-27 trimethylation (H3K27me3) are often associated with gene activation and repression, respectively [99,100].

**Figure 3:**
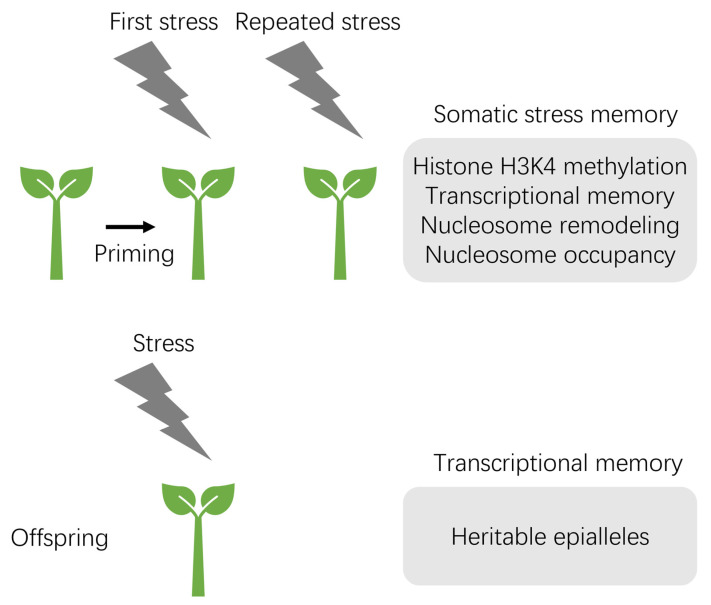
Epigenetic and chromatin-based stress memory in plants. Plants acquire stress memory after the initial stress exposure, known as priming, which can enhance their resistance to subsequent stress. Somatic stress memory is temporary. H3K4 hypermethylation, transcriptional memory, nucleosome remodeling, and nucleosome occupancy have been observed in somatic stress memory in response to abiotic stress. Transgenerational stress memory is passed on to stress-free offspring. Heritable epialleles with differential DNA methylation may be involved in the chromatin-based mechanisms of transgenerational stress memory.

A plant is primed by a transient biotic or abiotic stress and stores stress memory over a period of time to perform more quick and vigorous responses when stress recurs. Chromatin-mediated environmental memories are classified into somatic memory and transgenerational memory (Figure 3). Somatic stress memory occurs in only one generation of the organism, whereas transgenerational stress memory can be inherited by at least the first stress-free offspring generation [101]. Somatic stress memory is frequently associated with histone H3K4 methylation and nucleosome occupancy during priming [101]. For instance, the maintenance of H3K4me3 at two drought-inducible genes (RB29B and RAB18) after re-water treatment is essential for drought stress priming [98,102].

Mechanisms underlying intergenerational or transgenerational stress memory are far from clear. Epialleles with differential DNA methylation may be involved in the inheritance of chromatin-based stress memory [101]. In *Arabidopsis*, epigenetic alterations in DNA methylation are heritable across generations [103,104]. However, the DNA methylation state is predominantly stable within and across six successive generations and impervious to repeated drought stress [105]. Transgenerational effects or inherited stress memory to drought were not found in five *Arabidopsis* accessions under mild drought [49]. In contrast, the establishment of short-term stress memory related to DNA methylation was found in rice under recurrent drought stresses and recovery treatments [97,106]. Moreover, higher stability and inheritance of DNA methylome under water-deficit stress was observed in drought-tolerant lines than that of drought-sensitive lines in rice [107] and maize [56]. This raises the question of how drought-induced DNA methylation variation underpins transgenerational stress memory. Recent research on white clover [108] and strawberry [109] suggests that a portion of effects triggered by environmental interaction can be inherited, and population history contributes to a stable epigenetic memory in adaptation to drought.

## 9. Conclusions and Future Perspective

**Figure 4:**
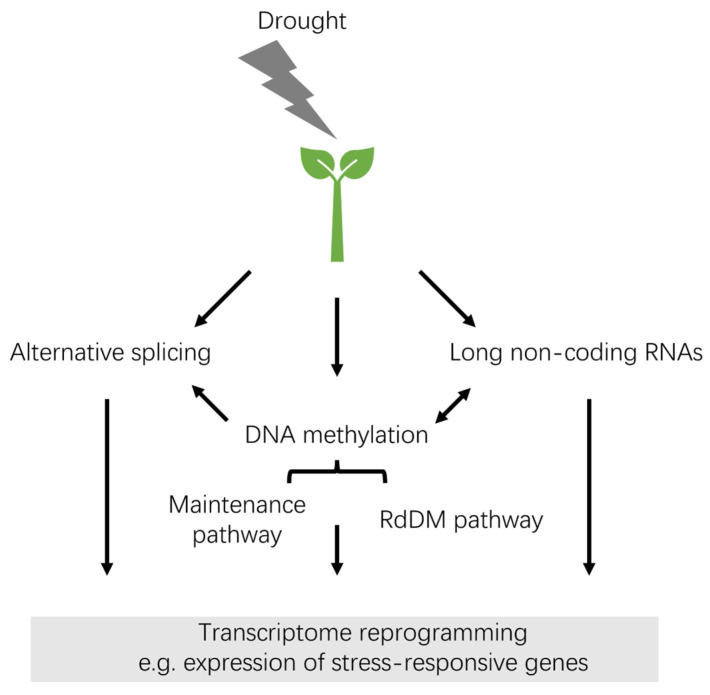
Role of DNA methylation in response to drought stress in plants. Drought stress induces DNA methylation modifications. DNA methylation is maintained by DNA methyltransferases and demethylases. De novo methylation can occur through the RdDM pathway in plants. Additionally, alternative splicing and long noncoding RNAs play a role in the response to drought stress. DNA methylation may influence alternative splicing events in stress response and interact with long noncoding RNAs. The modifications in DNA methylation, alternative splicing, and long noncoding RNAs contribute to the reprogramming of the transcriptome, including the expression of stress-responsive genes. RdDM, RNA-directed DNA methylation.

In this review, we have summarized recent studies on DNA methylation in various crops under drought stress (Table 1) and provided insights into the epigenetic response to drought stress (Figure 4). The changes in DNA methylation induced by drought stress vary in species-, genotype- and tissue-specific manners. Among the three DNA methylation contexts, the asymmetric CHH is hypersensitive to drought stress. Most available studies indicate an important role of DNA methylation in regulating the expression of stress-responsive genes. Additionally, RdDM-based DNA methylation potentially targets TEs and genes, silencing them in a sequence-specific manner in response to drought stress. AS events and lncRNAs, which play a crucial role in stress response, may be associated with DNA methylation under drought stress in some crops. However, further exploration of the epigenetic regulation of AS events and lncRNAs under abiotic stresses is necessary.

Several approaches are available for locus-specific manipulation of DNA methylation in plant genomes. One of these approaches is Virus-Induced Gene Silencing (VIGS) technology, which generates siRNAs that can activate the RdDM pathway to silence gene expression [110]. However, traditional methods for epigenetic modifications may result in global alterations in DNA methylation. Recently, epigenome editing technologies have been successfully applied for precise chromatin perturbations [111,112,113]. For example, the fusion protein of Tet1 or Dnmt3a with a catalytically inactive dCas9 has been shown to regulate the methylation status at the promoter region of a targeted reporter gene [112]. A set of epigenome editing tools, consisting of a catalytically inactive dCas9 fused with an optimized GCN4 tail and a library of nine effectors, has been developed for systematic chromatin modifications at specific loci [113]. These emerging epigenetic modification techniques will offer opportunities for epigenetic manipulation at targeted sites in crops to improve their agronomic traits.

The effects of climate change, such as global warming and low rainfall, have accelerated the prevalence of drought stress. There is an urgent necessity to develop climate-resilient crops that can adapt to unpredictable extreme weather conditions. Epigenetic variations are heritable changes that enable plants to cope with environmental stresses without altering DNA sequences. Understanding the connection between epigenetic changes and adaptation to drought stress will facilitate the molecular breeding of important crops. The epigenome, which includes DNA methylation, histone modification, and RNA-mediated gene silencing, should be considered as a whole system. With the advancements in high-throughput techniques such as second- and third-generation sequencing, RNA and chromatin immunoprecipitation (ChIP) sequencing, and single-cell sequencing, it will be easier to draw the epigenomic landscape of drought stress response at single-cell resolution. In the future, developing epigenetic modification techniques and gaining a deeper understanding of epigenetic mechanisms will offer a promising approach to developing crops with heritability and stability to enhance stress tolerance.

**Table 1:** Recent research on epigenetic modification in crops for drought stress.

Plant Species	Method ^a^	Key Findings	Refs
*Arabidopsis thaliana*	WGBS, RNA-seq	The *Arabidopsis* methylome is stable under transgenerational drought stress	[105]
	WGBS, RNA-seq	No inherited effects in DNA methylation to mild drought	[49]
*Brassica napus* (oilseed rape)	MSAP	Methylation changes among four generations in response to drought stress	[114]
*Camellia sinensis* (tea)		The expression of methyltransferase and demethylase genes in tea under abiotic stress	[38]
*Dendrobium huoshanense* (Mihu)	MSAP	Decreased DNA methylation and increased demethylation rate of methylated sites with treatment of exogenous NO and PEG	[115]
*Eucalyptus globules* (blue gum)	MS-RAPD	DNA methylation was increased over the dehydration period and rapid reduced after rehydration	[116]
*Fragaria nilgerrensis* (wild strawberry)	WGBS, RNA-seq	Differential correlation of CG, CHG and CHH with gene expression under drought stress	[59]
*Fragaria vesca* (woodland strawberry)	WGBS	Quantification of drought-induced changes in DNA methylation in strawberry population	[109]
	WGBS, RNA-seq	Correlation between DNA methylation, TEs and gene expression regulation under abiotic stresses	[64]
*Gossypium hirsutum* (cotton)	WGBS	CHH content is most sensitive to drought stress. lncRNAs may cause variations in methylation patterns	[46]
*Hibiscus cannabinus* (Kenaf)	MSAP	DNA methylation decreased in drought-tolerance hybrid. Knockdown of the DnaJ increased the drought sensitivity in seedling	[65]
*Hippophae rhamnoides* (sea buckhorn)	WGBS, RNA-seq	Increase in DNA methylation level by drought stress. Involvement of HrMET1 and HrDRM1 in drought-induced hypermethylation	[117]
*Hordeum vulgare* (barley)	MSRE-qPCR, siRNA-seq	Terminal drought stress induced DNA methylation at the HvCKX2.1 promoter through RdDM pathway	[67]
	Locus specific BS-seq	The DNA methylation status of the demethylase gene promoter induced by drought	[118]
	CRED-RA	Changes in methylation pattern of three barley cultivars under drought stress	[119]
*Linum usitatissimum* (linseed)	WGBS, RNA-seq	Regulation of methylation on alternative splicing under repeated drought stress	[50]
*Lolium pereme* (ryegrass)	MSAP	Decrease in DNA methylation due to drought exposure and identification of candidate genes	[53]
*Macrotyloma uniflorum* (horse gram)	MSAP	Methylation was higher in drought-sensitive line than that in drought-tolerant line	[120]
*Malus domestica* (apple)	WGBS, RNA-seq	The linkage of DNA methylation and gene expression in drought-sensitive and drought-tolerant cultivars	[40]
	Locus-specific BS-seq	Methylation in the promoter of drought-tolerance gene MdRFNR1 induced its expression	[68]
*Morus alba* (mulberry)	WGBS, RNA-seq	The contribution of CG content in response to drought stress	[41]
	MethylRAD	Changes in DNA methylome under drought stress	[121]
	RNA-seq	Identification of differentially expressed lncRNAs and mRNAs under drought stress	[94]
*Oryza sativa* (rice)	MSAP	Identification of site-specific DNA methylation induced by drought	[122]
	ChIP-Seq, RNA-seq	Identification of genes showing changes both in histone modification and expression under drought stress	[123]
	MSAP	The inheritance of epigenetic variations induced by drought stress	[107]
	WGBS, RNA-seq, siRNA-seq	Differences in DNA methylation, gene expression and smRNA abundance of three rice cultivars in response to drought	[72]
	MeDIP-seq, Mircoarray	Differential methylated region/genes in two rice cultivars under drought stress	[124]
	WGBS, ssRNA-seq	Detection of differentially expressed transcripts and lncRNAs under repeated drought stress	[97]
	MSAP	Differential methylation and gene expression associated with drought adaptation	[125]
	WGBS, RNA-seq	The correlation between hypomethylation in CHH context and higher expression of stress responsive genes	[55]
	WGBS	Identification of drought stress memory-related differentially methylated regions	[106]
	WGBS, RNA-seq	Differential methylation regions and expressed genes in two rice accessions for tolerance to drought stress	[126]
*P. deltoides* × *P. nigra*	HPLC	DNA methylation under changing water conditions depends on development and genotype	[127]
*P. deltoides*, *P. nigra* and *P. laurifolia* hybrids	Microarray	Epigenomic basis for transcriptome divergence in the clone history	[128]
*P. deltoides* × *P. nigra* hybrids	HPLC	The correlation between DNA methylation level and productivity	[129]
*P. tomentosa*	WGBS, RNA-seq	The relationship of DNA methylation diversity and drought resistance	[54]
*P. trichocarpa*	WGBS, RNA-seq	Unmethylated cis-splicing sites and methylated trans-splicing sites	[51]
*Populus* × *euramericana*	Microarray	Coordinated variations in DNA methylation and gene expression in reponse to water availability	[130]
*P. tremula* × *P. alba*	WGBS, RNA-seq	Improved drought tolerance in DDM1 RNAi lines	[45]
*Sesamum indicum* (sesame)	MSAP, RNA-seq	De novo methylation by drought stress and demethylation during the recovery phase	[131]
*Setaria italica* (foxtail millet)	BS-PCR	H_2_S signals may mediating DNA methylation on the promoter of six osmotic stress-responsive TFs	[132]
*Solanum lycopersicum* (tomato)		DNA methylation and histone modifications in an adaptive water stress-responsive gene	[133]
	WGBS, siRNA-seq	The controlling of siRNAs and the RdDM pathway on *Rider* activity	[71]
*Solanum melongena* (eggplant)		Dynamic expression of DNA methyltransferases and demethylases induced by drought stress	[36]
*Solanum tuberosum* (potato)		Response of five potato cultivars to methylation inhibitor and mannitol	[134]
	RNA-seq	Differential expression genes under drought stress and DNA methylation inhibitor treatment	[135]
*Triticum aestivum* (wheat)	MSAP	Identification of differential methylated TEs under drought stress	[136]
	MSAP	Higher demethylation in drought-tolerant genotype than that in drought-sensitive genotype after drought stress treatment	[58]
		Differential expression of DNA methyltransferases during drought stress	[37]
*Vicia faba* (faba bean)	MSAP	Decreased in the total methylation level in leaf due to drought exposure	[52]
*Vigna radiata* (mungbean)	WGBS, RNA-seq	Effects on CHH contexts by drought stress and genotypic variations	[57]
*Zea mays* (maize)	MSRE-qPCR, RNA-seq	DNA methylation at the ZmNAC111 promoter represses its expression and increases drought sensitivity	[66]
	AMP-PCR	Isolated and analyzed 18 differentially methylated fragments in seedling subjected to water deficit	[137]
	MSAP	Hypo- and hypermethylation of candidate genes induced by drought	[138]
	MeDIP, RNA-seq	Stable methylome of drought-tolerant lines. A positive correlation between gene body DNA methylation and splicing events	[56]

^a^ Methods for estimation of DNA methylation and gene expression are listed. Abbreviations: WGBS, Whole genome bisulfite sequencing; MSAP, Methylation-sensitive Amplification Polymorphism method; MeDIP-seq, Methylated DNA Immunoprecipitation sequencing; MSRE, Methylation-sensitive Restriction digest; MS-RAPD, Methylation-Sensitive-Random Amplified Polymorphic DNA; AMP-PCR, Amplified methylation polymorphism PCR; ChIP-Seq, Chromatin immunoprecipitation sequencing; CRES-RA, Coupled restriction enzyme digestion-random amplification; HPLC, High Performance Liquid Chromatography; RNA-seq, RNA sequencing; siRNA-seq, small interfering RNA sequencing; ssRNA-seq, whole-transcriptome strand-specific RNA sequencing.
